# Characteristics of PVDF Membranes Irradiated by Electron Beam

**DOI:** 10.3390/membranes5010001

**Published:** 2015-01-05

**Authors:** Babak Jaleh, Negin Gavary, Parisa Fakhri, Nakatan Muensit, Soheil Mohammad Taheri

**Affiliations:** 1 Department of Physics, Bu-Ali Sina University, Hamedan 65174, Iran; E-Mails: N.gavary@basu.ac.ir (N.G.); P.fakhri91@basu.ac.ir (P.F.); Sm.taheri91@basu.ac.ir (S.M.T.); 2 Department of Physics, Faculty of Science, Prince of Songkla University, Songkhla 90110, Thailand; E-Mail: nantakan.m@psu.ac.th

**Keywords:** electron beam irradiation, membrane, PVDF

## Abstract

Polyvinylidene fluoride *(*PVDF) membranes were exposed vertically to a high energy electron beam (EB) in air, at room temperature. The chemical changes were examined by Fourier Transform Infrared Spectroscopy (FTIR). The surface morphologies were studied by Scanning Electron Microscopy (SEM) and showed some changes in the pore size. Thermogravimetric (TGA) analysis represented an increase in the thermal stability of PVDF due to irradiation. Electron paramagnetic resonance (EPR) showed the presence of free radicals in the irradiated PVDF. The effect of EB irradiation on the electrical properties of the membranes was analyzed in order to determine the dielectric constant, and an increase in the dielectric constant was found on increasing the dose. The surface hydrophilicity of the modified membrane was characterized by water contact angle measurement. The contact angle decreased compared to the original angle, indicating an improvement of surface hydrophilicity. Filtration results also showed that the pure water flux (PWF) of the modified membrane was lower than that of the unirradiated membrane.

## 1. Introduction

Modification of polymers using irradiation is a common method in many researches to enhance the physical and chemical properties of polymers. Ionizing radiation induces changes in the chemical structure and physical properties of polymers. Such changes may arise from crosslinking, main chain scission, or evolution of hydrogen, depending upon the chemical and physical nature of the polymer and the type of radiation. Electron beam (EB) irradiation has long been used in fluoropolymers to enhance thermal stability and dielectric loss [1,2,3,4,5]. 

Poly(vinylidene fluoride) (PVDF) is a fluorinated polymer which possesses good physical and chemical properties. PVDF has been the focus of several studies and industrial uses due to good chemical resistance, biocompatibility and processability and its favorable electrical, thermal and mechanical properties [6]. It has also been widely studied in the filtration membrane field either in the form of porous membranes or as modified membranes [7]. 

Several researchers have worked on the investigation of the behavior of PVDF film under EB irradiation. Changes in crystallinity, melting point, gel fraction and crosslinking of PVDF film by EB irradiation have been reported [8,9,10,11]. Also, Hui-Jian Ye *et al.* [12] investigated the effect of electron irradiation on the electroactive phase and dielectric properties of PVDF films. The results show a reducing trend for the relative content of β-phase, crystallinity, dielectric constant and the maximum exothermal temperature of PVDF films with increasing doses.

One of the applications of PVDF is the use of PVDF membranes in water treatment. The major problem of PVDF membranes in this application is the hydrophobicity. Therefore it is very important to improve the hydrophilicity of PVDF membranes [13]. Also, PVDF has been observed to experience degradation during high temperature operations. Therefore, increasing the thermal stability of PVDF has made it an interesting membrane material for a wide range of industrial applications [14]. Moreover, PVDF provides remarkable electrical properties, such as great dielectric performance, that is essential in applications on energy conversion and storage [13]. For these reasons, the investigation of the hydrophilicity, as well as the thermal and electrical properties of PVDF under EB irradiation is highly desirable, which requires further studies. This is in spite of the fact that different irradiation conditions (energy, dose rate,* etc.*) on various forms of PVDF (film, membrane,* etc.*) may lead to different results. The goal of this research work was to investigate the structural, thermal, electrical, morphological, and hydrophilicity changes induced in PVDF membranes by EB irradiation. In this work, PVDF membranes were initially irradiated by 10 MeV high dose rate up to 630 kGy/min at different doses, ranging from 50 to 300 kGy in an air atmosphere. Here, some characterization techniques via Fourier Transform Infrared Spectroscopy (FTIR), scanning electron microscopy (SEM), thermogravimetric analysis (TGA), electron paramagnetic resonance (EPR), LCR meter, water contact angle measurement, gel fraction determination and pure water flux were exploited to understand the induced changes in the physico-chemical properties of the irradiated membrane. Finally the EB induced PVDF as a membrane was investigated to show how the hydrophilicity degree of the membrane changes as a function of EB dose in order to fabricate a biocompatible material.

## 2. Experimental Details

Poly(vinylidene fluoride) membrane was purchased from Millipore (Dublin, Ireland) with a 0.22 μm pore size. Membranes were irradiated by the Rhodtron TT200 type (IBA Company, Louvain-La-Neuve, Belgium). The nominal electron energy was 10 MeV/electron. The beam current was kept constant at 7 mA during the experiments. The samples were placed on a conveyor belt. The clearance between the accelerator output windows and the conveyor was ~120 cm. The samples traversed under a scanning horn several times so as to receive the desired dose. Irradiation was carried out in air at room temperature. The dose rate was 10 kGy/s corresponding to 25 kGy per pass. The samples were irradiated on an aluminum pallet for 25 kGy in each cycle of irradiation and the cycle time was 7–8 min, thus the temperature did not increase significantly; about 40 °C. The absorbed dose was selected in the range of 50–300 kGy. The chemical changes which took place in electron irradiated PVDF at doses up to 300 kGy were studied by FTIR, at a wave number range from 400 to 4000 cm^−1^ using a Shimadzu FTIR spectrophotometer 8000 (Shimadzu, Tokyo, Japan). The surface morphology of membranes was analyzed by SEM (Cam scan MV2300, TSCAN, Czech Republic). TGA was carried out using a Perkin Elmer system. The temperature range was from room temperature up to 800 °C at a heating rate of 10 °C/min under a nitrogen flow of 70 cm^3^/min. Moreover, a Bruker EMS104 EPR analyzer (Bruker Rheinstetten, Karlsrush Karlsruhe, Germany) at 9.8 GHz microwave frequency was exploited to detect the free radical signal spectrum. The major specifications of the analyzer are 12.5 mW microwave power, sweep width 50 G, 10.5 s, modulation amplitude 8.02 G, sweep time filter time constant 20.5 ms and receiver gain 30 dB. A precision LCR meter Agilent 4258A (Hewlett Packard, Palo Alto, CA, USA) with a 16,451 b dielectric test fixture at various frequencies was exploited to determine the dielectric constants for the irradiated and unirradiated PVDF membranes. Surface hydrophilicity of the membranes was evaluated using contact angle measurements with a Kruss G10 goniometer (Kruss, Hamburg, Germany) equipped with image analysis software. Deionized water was dropped onto the sample from the needle of a microsyringe during the test. A picture of the drop was captured after the drop settled onto the sample. The contact angles could be calculated by the software by analyzing the shape of the drop. The contact angle θ was an average of five measurements. After irradiation, the PVDF membranes were weighed (W_i_). The gel fraction was determined by extraction with N,N-dimethylacetamide (DMAC) at 167 °C for 24 h. The insoluble portions of the membranes, which consisted of crosslinked PVDF, were dried then weighed (W_d_). The gel fraction is defined [11] as

(1) $$\text{Gel fraction (\%)}=\frac{{\text{W}}_{\text{d}}}{{\text{W}}_{\text{i}}}\times 100$$

To investigate the filtration performance of the samples, the membranes were used to determine the pure water flux (PWF) at a trans-membrane pressure (TMP) of 1.0 bar.

The permeability was measured under steady state flow. PWF was calculated as follows:

(2) $${\text{J}}_{\text{w}}=\frac{\text{Q}}{\text{A }\cdot \Delta \text{t}}$$

where J_w_ is the pure water flux (L/m^2^h); Q the quantity of filtrate collected (L); Δt the permeate time (h); and A is the membrane area (m^2^).

## 3. Results and Discussion

**Figure 1:**
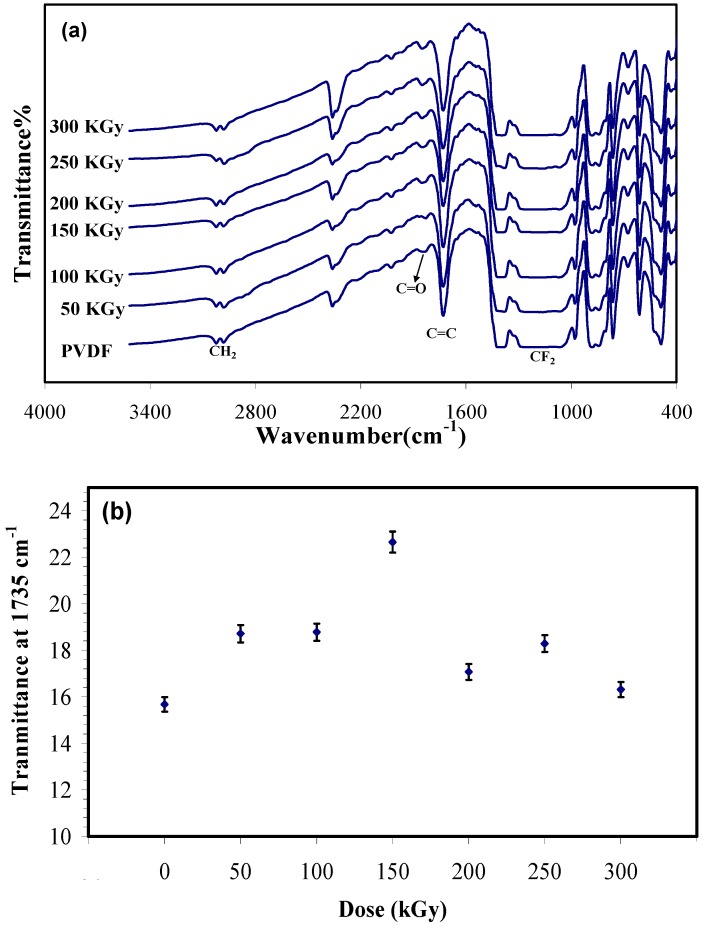
(**a**) Fourier transform infrared spectroscopy (FTIR) spectra of irradiated and unirradiated Polyvinylidene fluoride *(*PVDF) membrane; and (**b**) absorbance at 1735 cm^−1^ (C=C) versus electron beam (EB) dose in terms of kGy.

Figure 1a shows FTIR spectra of the unirradiated and irradiated PVDF membranes. In the unirradiated sample, two absorption band located at 2978 cm^−1^ and 3024 cm^−1^ correspond to the symmetric and asymmetric stretching vibration of the CH_2_ groups. The absorption region from1300 to 1000 cm^−1^ corresponds to fluorocarbon absorption. The band appearing at 1735 cm^−1^ is due to the C=C stretching vibration and the peak at 1828 cm^−^^1^ is assigned to the C=O (carbonyl) groups resulting from the formation of hydroperoxide radicals initiated by irradiation in air [11,15,16]. The spectra of the irradiated membrane did not show any major changes in the main absorption bands relative to those of the unirradiated sample. This result has been reported in other works before [8,9,10,11]. However, a difference was observed between unirradiated and irradiated samples that is shown in Figure 1b. This chart shows the transmittance at 1735 cm^−1^ as a function of dose. It illustrates by increasing the dose to 150 kGy the transmittance increases (it means that the intensity of the peak decreases) that is resulting from crosslinking, then the transmittance decreases as the irradiation dose goes higher, which can be attributed to the predomination of chain scission over crosslinking at higher doses (>150 kGy). 

**Figure 2:**
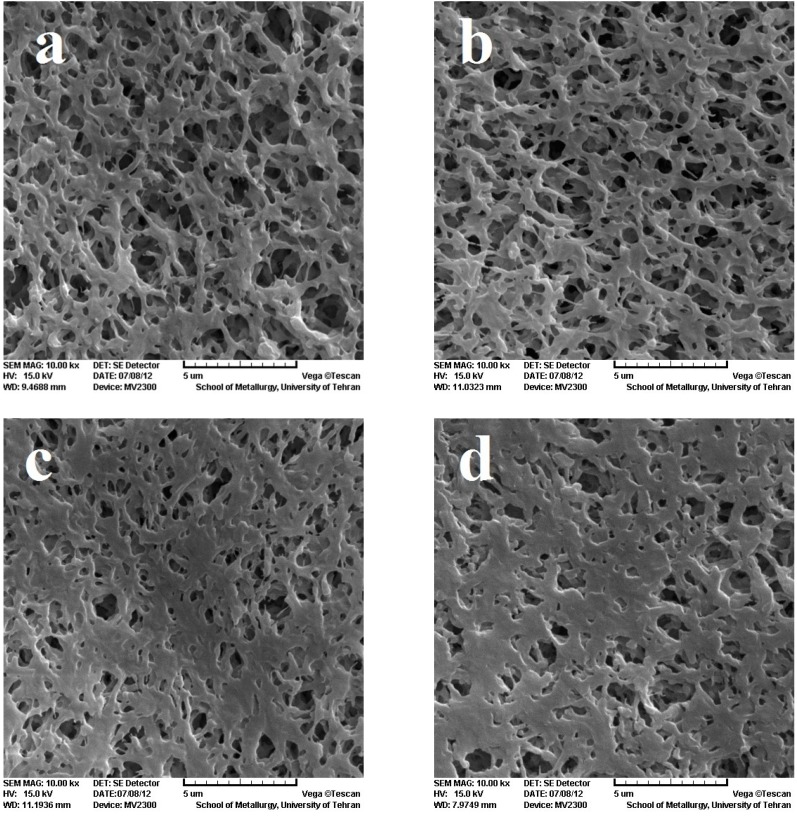
Scanning electron microscopy (SEM) image of PVDF membrane. (**a**) reference;(**b**) 100 kGy; (**c**) 150 kGy; (**d**) 300 kGy.

To investigate the surface morphology, SEM was employed to compare the effect of the EB irradiation on the PVDF membrane, as shown in Figure 2. The figures show the pore size of the modified membranes decreases on increasing the dose. The explanation for this observation can be related to the fact that irradiation causes crosslinking in the polymer, forming chemical bonds between chains, which leads to the decrease in pore size [7]. Reduction of the size of the pores, increases PVDF membrane application as a filter for smaller particles. 

**Figure 3:**
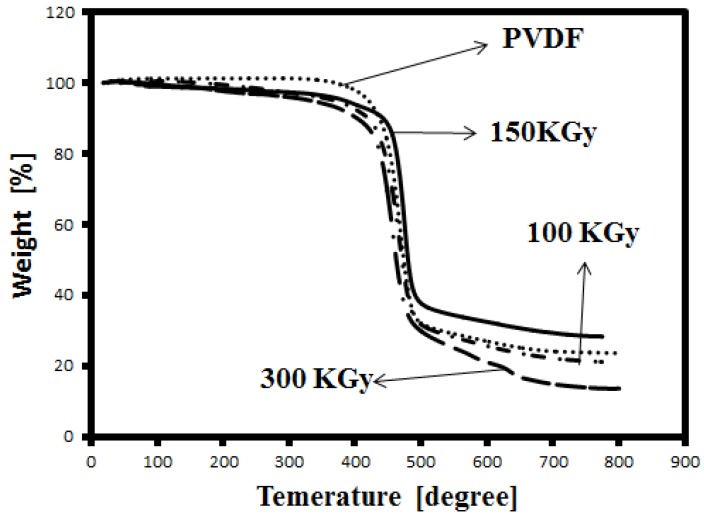
Thermogravimetric analysis (TGA) curve of irradiated and unirradiated PVDF membrane.

Thermal stability of the membranes was studied by TGA. Figure 3 shows the TGA thermograms of unirradiated and irradiated PVDF membranes. The thermo-degradation of PVDF is observed around 420 °C (determined from the first inflection point of the curve). By increasing the dose, the degradation temperature increases to 425 and 460 °C for 100 and 150 KGy, respectively. Indeed, irradiation at 150 KGy causes an increase of the degradation temperature of about 40 °C. Therefore, the irradiation modifies the thermal stability of the membrane. The resistance of the membrane toward thermal degradation may be related to the crosslinking in the samples. As shows in Figure 3, the degradation temperature decreased to about 428 °C, indicating that in higher doses crosslinking decreases. These results are in agreement with the FTIR results.

**Figure 4:**
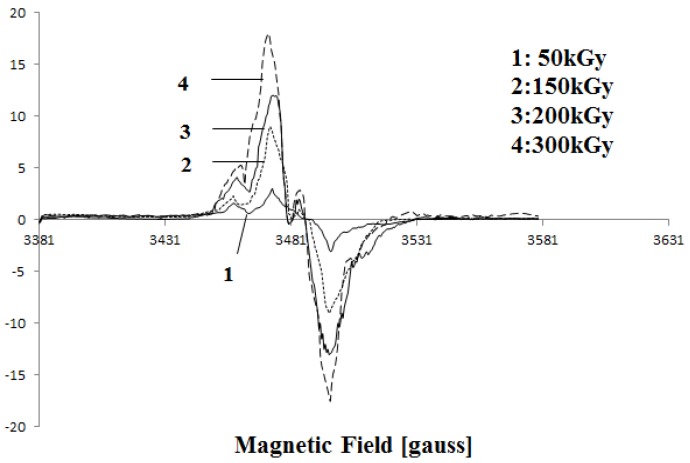
Electron paramagnetic resonance (EPR) curve of irradiated PVDF membrane by 50 kGy, 200 kGy and 300 kGy.

The formation of free radicals was detected by EPR measurements. Free radicals induced in PVDF by ionizing radiation have been previously studied. EB irradiation of PVDF leads to a scission of C-H, C-F, and C=C bonds and creation of –˙CF_2_ , –˙CH_2_ , –CF_2_–˙CH–CF_2_– and –CH_2_–˙CF–CH_2_– radicals. Some of the radicals become oxygenated in the air and alkoxy or alkyl peroxy radicals appear [10,17]. The radical amount is proportional to the absorbed dose. Figure 4 shows changes in the EPR spectra of PVDF after irradiation with various doses. From the Figure it can be seen that the intensity of the peaks increases with increasing dose. This confirms the increasing number of free radicals at higher doses. Also, a peak appears around 3480 G above 150 kGy, this peak is probably related to peroxide radicals [17].

**Figure 5:**
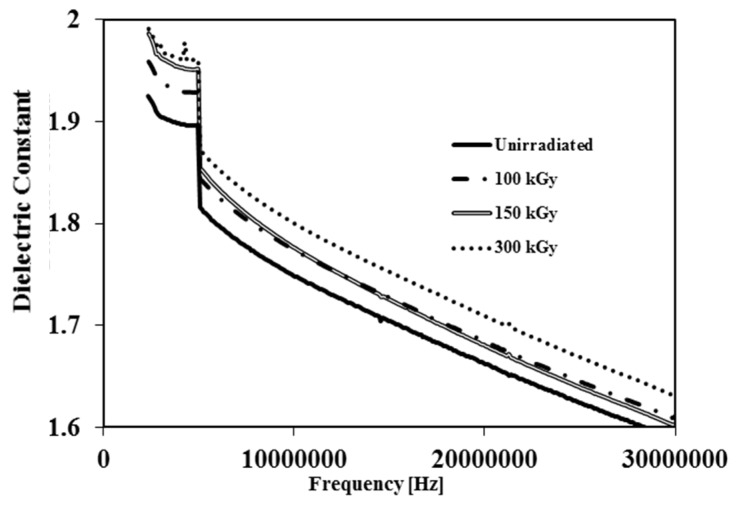
Dielectric constant *vs.* frequency of unirradiated and irradiated PVDF membranes by 100 kGy, 150 kGy and 300 kGy.

The dielectric constant is a measure of the ability of a material to store electrical energy. Figure 5 displays the dielectric constant *vs.* frequency for irradiated and unirradiated membranes. It is clear from Figure 5 that irradiation leads to a gradual increase of dielectric constant as the amount of dose is increased. The increased dielectric constant at higher doses is related to a decrease in membrane void volume. Whereas air has a low dielectric constant*,* by reduction of void volume, the air content inside samples decreases resulting in an increase in the dielectric constant [18]. On the contrary Hui-Jian Ye *et al.* observed that the dielectric constant of PVDF film decreased by increasing the EB dose since the samples they used were films rather than membranes [12].

**Figure 6:**
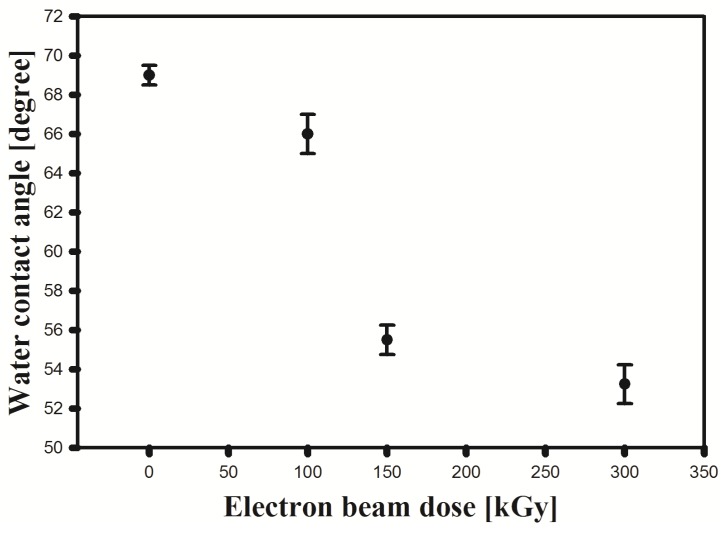
The water contact angle of irradiated and unirradiated PVDF membrane.

The water contact angle is often used to evaluate the hydrophilicity of the membranes. Figure 6 illustrates the variation of the contact angle with the membrane irradiation dose. As shown, the contact angles of the membrane decreased with increasing irradiation dose. The water contact angle for PVDF was 69°. By irradiation of PVDF, the contact angle decreased to 53°, which may be ascribed to both the production of hydrophilic radicals and hydroperoxides, as well as surface rugosity changes [19]. This result indicates that EB irradiation improves the surface hydrophilicity of a PVDF membrane. Also, because of the importance of hydrophilicity in filtration, the PVDF application in this field should increase.

**Figure 7:**
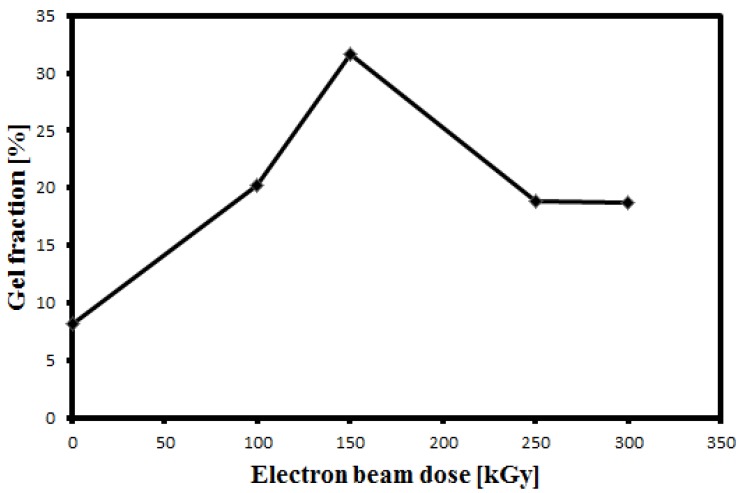
Gel fraction as a function of radiation dose.

Since sufficient cross-linking to cause polymer bulk gelation is evidenced by drastic changes in solubility and physico-mechanical behavior, the degree of crosslinking may be determined by the measurement of “gel content” [15]. The gel content of the irradiated sample was measured to quantify the amount of crosslinking induced by irradiation. Figure 7 shows the variation of the gel fraction with the irradiation dose. It was found that the gel fraction increased up to 150 kGy which is due to crosslinking. This is in agreement with the result of the effect of EB irradiation on PVDF film reported in [11]. Conversely, the gel fraction decreases corresponding to the decrease in crosslinking to a constant value at doses greater than 200 kGy. This result is in agreement with the finding of FTIR and TGA analyses.

**Figure 8:**
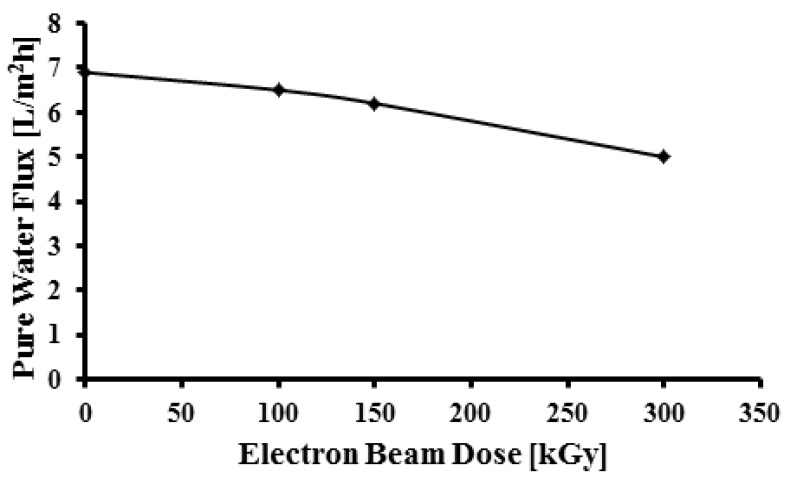
Pure water flux as a function of radiation dose.

As shown in Figure 8 the pure water flux of the porous membranes decreased on increasing the dose which is attributable to the changes in the surface porosity of the membrane. This result is in agreement with SEM images. Often the increase in hydrophilicity should lead to higher and not lower water flux, but here the increase of hydrophilicity is related to roughness and the functional groups. On the other hand irradiation resulting in crosslinking and hence reduction of pore size leads to reduction of water flux.

## 4. Conclusions

The irradiated PVDF by EB from 50 to 300 kGy in an air atmosphere was investigated using instrumental analysis techniques such as, FTIR, SEM, TGA, EPR, LCR meter and contact angle to study the physico-chemical properties of the polymer. FTIR shows that the absorption bands of the irradiated polymer do not change significantly, and remain similar to the unirradiated PVDF. However, transmittance of C=C band increased at low dose to 150 kGy, suggesting the formation of crosslinked structures. In TGA thermograms the decomposition temperature becomes higher at larger doses. This result indicates that the thermal stability of the membrane increases. EPR measurement confirmed the increasing number of free radicals at higher doses. The effect of electron beam irradiation on porous membrane leads to smaller pores, that may lead to the development of PVDF membrane as a filter for smaller particles. Reduction of the void volume resulted in an increase of the dielectric constant on increasing dose. The water contact angle decreased on increasing the dose because of oxidation and the formation of polar groups on the surface of the membrane, indicating an improvement in hydrophilicity of the membrane. The pure water flux of the membranes decreased on increasing the dose due to decreasing pore size, confirming that the filterability properties had been improved.
